# Supplementary material for: A sustainable yarn tension control technique for optimizing textile dyeing efficiency and water use
Source: PLoS One. 2026 May 15;21(5):e0348871. doi: 10.1371/journal.pone.0348871 (PMC13178881; doi:10.1371/journal.pone.0348871)
Supplement: Attachment — Submitted filename: Response.pdf [file pone.0348871.s002.pdf]

<u>Response to Editor and Reviewers</u>

A Sustainable Yarn Tension Control Technique for Optimizing Textile Dyeing Efficiency and Water Use

<u>PONE-D-25-48765</u>

The authors would like to seize this chance to thank the editor and reviewers for their appreciated effort and time. The authors respond to all the raised comments, attempting to fulfill all the requirements through this stage. The authors hope that their reply receives considerable satisfaction and also would like to clarify their sincere intention to make every effort/modification required to attain the reviewers' satisfaction and raise the quality of the submitted manuscript to fit with the journal's high standards.

<u>Journal Requirements:</u>

1. **Please ensure that your manuscript meets PLOS ONE's style requirements, including those for file naming. The PLOS**
   Author comment: Thank you for pointing out this.
   Author responses: We have carefully revised the manuscript to comply with PLOS ONE author guidelines and formatting requirements.
   We also restructured the manuscript to include Abstract, Introduction, Literature Review, Case Study, the Proposed Technique, Materials and Methods, Results and Discussion, Conclusions, Author contribution, Data availability, Funding and References.

2. **Please note that PLOS One has specific guidelines on code sharing for submissions in which author-generated code.**
   Author comment: We thank the reviewer for this comment
   Author responses: We confirm that this study did not involve any author-generated code. Therefore, PLOS ONE's code-sharing requirements are not applicable.

3. **In accordance with PLOS ONE's code sharing guidelines, the author-generated code has been provided as supplementary material, and the Data Availability statement has been revised to clearly describe how the code can be accessed.Please provide a complete Data Availability Statement in the submission form, ensuring you include all necessary access**
   Author comment: We thank the reviewer for this clarification.

Author responses: The data availability statement has been added at the end of the manuscript.

4. **Please ensure that you refer to Figures 4 and 11 in your text as, if accepted**
   Author comment: We thank the reviewer for this comment
   Author responses: The manuscript has been revised to explicitly refer to Figures 4 and 11 at the appropriate locations in the text.

5. **If the reviewer comments include a recommendation to cite specific previously published works, please review and evaluate these publications to determine whether they are relevant and should be cited. There is no requirement to cite these works unless the editor has indicated otherwise**
   Author comment: Thank you for this guidance.
   Author responses: The reviewers didn't recommend adding any further references.

<u>REVIEWER #1 COMMENTS</u>

Reviewer 1 comment #1: The manuscript is not organized according to this set of guidelines; this must be addressed.

Author comment: We thank the reviewer for pointing out.

Author Response: We have completely reorganized the manuscript to follow the standard PLOS ONE standard structure guidelines including abstract Introduction, literature review, Case Study, the Proposed Technique, Materials and Methods, Results and Discussion, Conclusions, Author contribution, Data availability, Funding and References.

Reviewer 1 comment #2: The authors should summarize the state of the art and highlight the relevance of the work submitted in the Introduction section. In the present form it is too broad, and not fully focused, e.g., the authors digress too much on general matters related to cleaner production, the introduction should be more focused.

Author comment: We thank the reviewer for pointing out

Author Response: The Introduction section has been revised to better summarize the current state of the art and to clearly highlight the relevance and novelty of the present study. The introduction is more focused on specific connection between yarn tension and water efficiency. Therefore, three subheadings have been added as follows: (1) water consumption in dyeing, (2) yarn tension's role in quality, and (3) the research gap connecting these two

Section 1.1: Water and Chemical Consumption: Which focuses on specific water and chemical volumes in dyeing

Section 1.2: Yarn Tension in Rewinding Process which explicitly connected yarn tension to water consumption

Section 1.3: Research Gap and Study Objectives: Clearly bounded the scope

Reviewer 1 comment #3: Some statistical treatment of the data is strongly advised, to highlight if the observed differences are statistically meaningful, e.g., for data in Table 2, how many replicates were performed, what was the standard deviation of the water use, and variation in dyeing uniformity?

Author comment: We thank the reviewer for this valuable suggestion.

Author response: The manuscript has been revised to add the missing statistical analysis data in the following:

1. We have clearly clarified Imperfection Index (IPI) and added the Standard Deviation in Table 1 and illustrated that we performed five replicates.
2. We have also clarified that the data presented in table 2 is the average of 10 dyeing batches and added the Standard Deviation for the Dye Uniformity data.
3. Two-sample t-test confirmed a statistically significant improvement ($p < 0.01$) in dye uniformity in line 378-380.

Reviewer 1 comment #4: The authors refer and present data (Table 3) of a life cycle analysis (LCA), but they do not present detailed information in materials and methods, e.g., scope, system boundaries, inventory data used, software used, uncertainty and sensitivity, which ISO guidelines were followed.

Author comment: We thank the reviewer for this valuable suggestion.

Author Responses: We first have added materials and method section. In this section we have added a subsection named Life Cycle Assessment (LCA) describing the scope, system boundaries, data used, and ISO guidelines.

Reviewer 1 comment #5:Minor glitches: "Furthermore, water Demand" should be "Furthermore, water demand"

Author comment: We thank the reviewer for pointing this out.

Author Response: The manuscript has been corrected so that "Furthermore, water Demand" now reads "Furthermore, water demand" in line 49.

Reviewer 1 comment #6: Mind that reference 14 is the pdf version of a powerpoint presentation prepared by the Institute of Environmental Engineering, Kaunas University of Technology, Lithuania and sponsored by the Sponsored by UNEP, Division of Technology, Industry, and Economics, with the title: INTRODUCTION TO CLEANER PRODUCTION (CP) CONCEPTS AND PRACTICE. The authors should consider using as reference a peer-reviewed paper focused also on cleaner production goals, strategies and implementation, e.g., DOI: 10.1002/csr.2693, https://doi.org/10.3390/su15032161, ...

Author comment: We thank the reviewer for this helpful suggestion.

Author Response: We have replaced the PowerPoint reference with the appropriate peer-reviewed publications, reference number [14].

<u>Reviewer #2 Comments</u>

Reviewer #2 comment # 1: The authors' claim of achieving 65–70% water savings and approximately 20% energy reduction during dyeing appears to rely primarily on manufacturer-reported liquor ratios rather than controlled experimental measurements, which undermines the validity of the conclusion.

Author comment: We thank the reviewer for this helpful suggestion.

Author Response #1: We stated in the "Results and Discussion" section that the water savings are based on an indirect result from the manufacturer optimized package density in the case study that enables the user to consume a lower liquor ratio (1:5 vs 1:15), then supporting this data by citing the theoretical principle that uniform package density reduces liquor ratios referring to [27,32].

Author Response #2: We have added a statement in the "**conclusion and future Recommendations**" clarifying that expanding experimental validation in controlled dyeing trials is recommended.

Reviewer #2 comment # 2: The sections discussing environmental impact and wastewater characteristics are well referenced; however, they are not clearly connected to yarn tension control, resulting in a diluted and unfocused contribution.

Author comment: We thank the reviewer for pointing this out

Author Response #1: We have strengthened this bonded link through the manuscript in the "yarn tension control and its impact on yarn quality and resources efficiency" section.

Author Response #2: We highlighted that this relation is also explained in line 503-512.

Reviewer #2 comment # 3: The materials and methods section require clearer and more systematic description to ensure reproducibility and transparency.

Author comment: We thank the reviewer for this important comment.

Author Response: The Materials and Methods section has added before the Results section, detailing the experimental setup, yarn quality measurement, industrial performance measurement, and LCA methodology.

Reviewer #2 comment # 4: The authors are strongly encouraged to provide directly measured water and energy consumption data, rather than inferring savings solely from liquor ratio values.

Author comment: We thank the reviewer for pointing this out.

Author Response # 1: We have clarified this response by adding a paragraph highlighted in line 503-512.

Author Response # 2: We have added a strong recommendation in the future work section recommending a controlled experiment to address the need for direct measurements according to the valuable advices of the reviewer in line 589- 606.

Reviewer #2 comment # 5: It must be clarified whether dyeing recipes, dye classes, batch sizes, and machine types were held constant across all experiments, as these variables can significantly influence the results.

Author comment: We thank the reviewer for pointing this out.

Author Response: We have added in the Materials and Methods a subtitle named as Industrial Performance Measurement that the comparison was a controlled industrial trial where the only variable was the rewinding tension. All other dyeing parameters (recipe, dye class, batch size, and machine type) were held constant between the "Before" and "After" conditions.

Reviewer #2 comment # 6: The manuscript should report the number of dyeing batches tested, along with variance measures and statistical significance to support the reliability of the findings.

Author comment: We thank the reviewer for pointing this out.

Author Response #1: We have clarified that 10 dyeing batches were tested for the industrial performance data (Table 2).

Author Response #2: We have added the Standard Deviation (SD) for the Dye Uniformity and stated that a two-sample t-test confirmed the statistical significance of the improvement ($p < 0.01$)

Reviewer #2 comment # 7. The IPI analysis appears to be based on only three trials per yarn count, yet no confidence intervals or statistical tests are provided to evaluate consistency or uncertainty.

Author comment: We thank the reviewer for pointing this out

Author Response: We have added the missing data regarding the statistical analysis replicate number which is five replicates and added the Standard Deviation (SD) for the Total IPI in table

Reviewer #2 comment # 8. Although the authors suggest broad industrial applicability, the study was conducted solely on Egyptian cotton (Giza 86) within a single manufacturing facility, limiting the generalizability of the conclusions.

Author comment: We thank the reviewer for this suggestion. We want to clarify that this manuscript is validating the authors previous work on tension control for man-made polyester yarn that has referred in [44]. Moreover, the current study has been implemented in a totally different raw material specifically Giza 86 variety. Results from EMTB control methodology demonstrate effectiveness in previous and current studies, although the raw material is totally different. Consequently, we can conclude that this approach is generalized to different yarn types. Only, the settings parameters (e.g., reference tension set points, speed limits, and transition thresholds) require adjustment for different yarn characteristics and machine configurations.

Author Response: We have added a statement in the Conclusions section acknowledging that the generalizability and explicitly serve as a strong proof-of-concept for the Global South's textile sector. We also recommend future work to validate the findings across a broader range of cotton types and industrial settings.

Reviewer #2 comment # 9: The manuscript frequently blends prior literature, factory-reported data, and experimentally measured results without clear distinction, which affects objectivity and makes it difficult to assess the source and reliability of specific claims.

Author comment: We thank the reviewer for pointing this out.

Author Response: We have created a Materials and Methods section to distinguish between the experimental IPI data and the industrial operational data.
